# 2-Phenyl-8,9,10,11-tetra­hydro-1-benzo­thieno[3,2-*e*][1,2,4]triazolo[1,5-*c*]pyrimidine

**DOI:** 10.1107/S1600536811007331

**Published:** 2011-03-05

**Authors:** Shridhar I. Panchamukhi, Nikhath Fathima, I. M. Khazi, Noor Shahina Begum

**Affiliations:** a Department of Chemistry, Karnatak University, Dharwad 580 003, India; b Department of Studies in Chemistry, Bangalore University, Bangalore 560 001, India

## Abstract

In the title compound, C_17_H_14_N_4_S, the benzothieno moiety is fused at one end of the pyramidine ring while the triazole ring with a phenyl substituent is fused at the other side. The triazole ring is almost planar [maximum deviation = 0.0028 (3) Å] while the cyclo­hexane ring adopts a half-chair conformation. In the crystal, pairs of inter­molecular C—H⋯N hydrogen bonds form centrosymmetric head-to-head dimers, corresponding to an *R*_2_^2^(8) graph-set motif. Further C—H⋯N inter­actions generate a zigzag chain of mol­ecules along the *c* axis. The supra­molecular assembly is consolidated by π–π stacking inter­actions [centroid–centroid distance = 3.445 (4) Å].

## Related literature

For the biological activity of thio­phenes, benzothio­phenes, pyrimidines and triazolopyrimidines, see: Shishoo & Jain (1992 ▶); Bradbury & Rivett (1991 ▶); Elslager *et al.* (1981 ▶); Yunosov *et al.* (1966 ▶); Blain *et al.* (1982 ▶). For related structures, see: Akkurt *et al.* (2008 ▶); Buzykin *et al.* (2008 ▶); Harrison *et al.* (2006 ▶); Lipson *et al.* (2006 ▶); Belcher & Squattrito (2006 ▶). For hydrogen-bond motifs, see: Bernstein *et al.* 1995 ▶). For puckering and asymmetry parameters, see: Cremer & Pople (1975 ▶); Nardelli (1983 ▶).
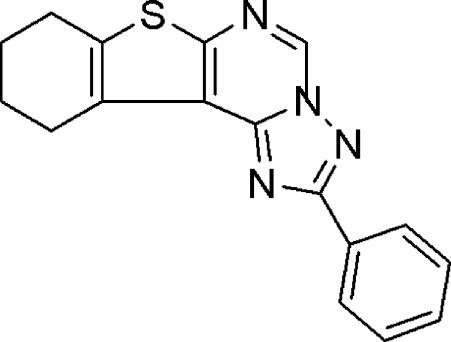


## Experimental

### 

#### Crystal data

C_17_H_14_N_4_S
*M* *_r_* = 306.38
Monoclinic, 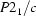

*a* = 8.6239 (16) Å
*b* = 20.512 (4) Å
*c* = 8.5952 (16) Å
β = 111.975 (3)°
*V* = 1410.0 (5) Å^3^
*Z* = 4
Mo *K*α radiation
μ = 0.23 mm^−1^
*T* = 296 K
0.18 × 0.16 × 0.16 mm

#### Data collection

Bruker SMART APEX CCD detector diffractometer
Absorption correction: multi-scan (*SADABS*; Bruker, 1998 ▶) *T*_min_ = 0.960, *T*_max_ = 0.964
8272 measured reflections
3042 independent reflections
2345 reflections with *I* > 2σ(*I*)
*R*_int_ = 0.057

#### Refinement

*R*[*F* ^2^ > 2σ(*F* ^2^)] = 0.060
*wR*(*F* ^2^) = 0.202
*S* = 1.25
3042 reflections
199 parameters
H-atom parameters constrained
Δρ_max_ = 0.72 e Å^−3^
Δρ_min_ = −0.66 e Å^−3^

### 

Data collection: *SMART* (Bruker, 1998 ▶); cell refinement: *SAINT-Plus* (Bruker, 1998 ▶); data reduction: *SAINT-Plus*; program(s) used to solve structure: *SHELXS97* (Sheldrick, 2008 ▶); program(s) used to refine structure: *SHELXL97* (Sheldrick, 2008 ▶); molecular graphics: *ORTEP-3* (Farrugia, 1997) ▶ and *CAMERON* (Watkin *et al.*, 1996) ▶; software used to prepare material for publication: *WinGX* (Farrugia, 1999 ▶).

## Supplementary Material

Crystal structure: contains datablocks global, I. DOI: 10.1107/S1600536811007331/pb2056sup1.cif

Structure factors: contains datablocks I. DOI: 10.1107/S1600536811007331/pb2056Isup2.hkl

Additional supplementary materials:  crystallographic information; 3D view; checkCIF report

## Figures and Tables

**Table 1:** Hydrogen-bond geometry (Å, °)

*D*—H⋯*A*	*D*—H	H⋯*A*	*D*⋯*A*	*D*—H⋯*A*
C5—H5⋯N2^i^	0.93	2.50	3.413 (3)	166
C11—H11*B*⋯N2^ii^	0.97	2.82	3.653 (5)	144

Symmetry codes: (i) 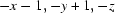; (ii) 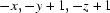.

**Fig. 1.**
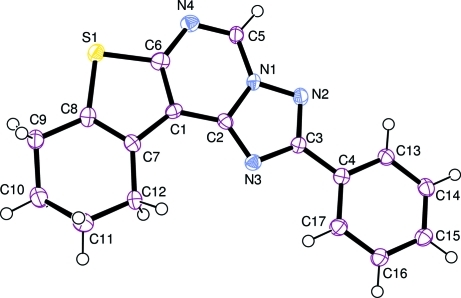
ORTEP (Farrugia, 1997) view of the title compound, showing 50% probability ellipsoids and the atom numbering scheme.

**Fig. 2.**
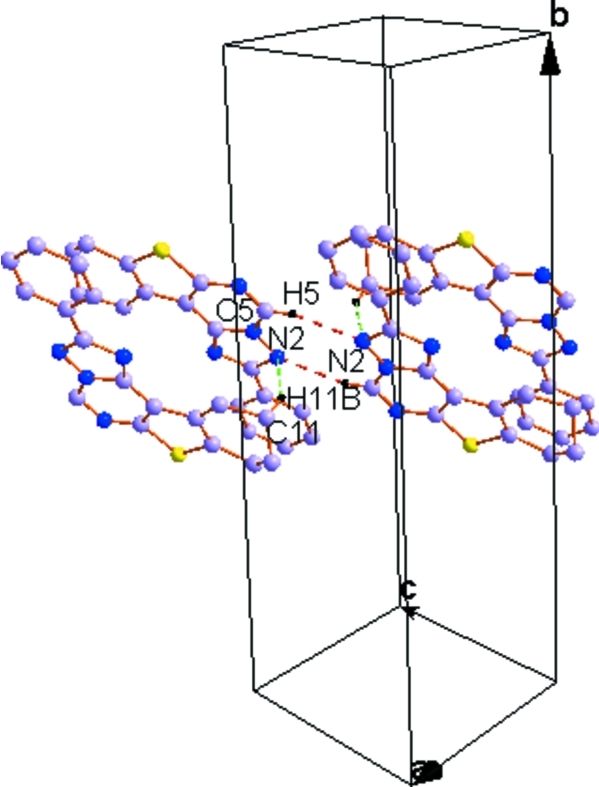
A unit cell packing of the title compound showing intermolecular interactions with dotted lines. H-atoms not involved in hydrogen bonding have been excluded.

## supplementary crystallographic information

### Comment

The Chemistry of thiophenes and benzothiophenes is well documented in the literature and has drawn much attention because of their wide spectrum of biological activities (Shishoo *et al.*, 1992). Pyrimidines and triazolopyrimidines are also associated with diverse biological activities (Bradbury *et al.*, 1991; Elslager *et al.*, 1981). In view of the pharmacological significance of thiophene and pyrimidine derivatives in well known drugs such as coramine (Yunosov *et al.*, 1966), antipyrine (Blain *et al.*, 1982) and also in continuation of our work on biologicallyactive nitrogen and sulfur heterocycles. In the title compound, the benzothieno moiety is fused at one end of the pyramidine ring and the triazole ring with a phenyl substituent is fused at the other side. The fused triazole-pyrimidine-benzotheino and the phenyl ring is coplanar with the dihedral angle 2.584 (3)°. The triazole ring is essentially planar similar to those reported earlier (Belcher & Squattrito, 2006; Buzykin *et al.*, 2008) with maximum deviation of atomsfrom their mean statistical planes being 0.0028 (3) Å. The N(1), atom of the triazole ring is in planar trigonal configuration similar to those reported earlier (Lipson *et al.*, 2006). The N(1)—N(2) bond length in the triazole ring is shorter {1.362 (4)Å} than the distance characteristic of a single N—N bond (1.47 Å). The N—C and N—N distances in the triazole ring vary from 1.36 (2) Å to 1.38 (4) Å respectively. The cyclohexene ring is in half-chair conformation. The plane calculation shows that the atoms C10 and C11 deviate from the mean plane C7/C8/C9/C12 constituting the ring by -0.358 (4)Å and 0.302 (4) Å,respectively, indicating that the conformation of the ring is that of a half-chair, with the atoms C10 and C11 being displaced by this overall planarity of the rest of the ring. The ring puckering parameters for the cyclohexene ring in the title compound are Q(2) = 0.3816 (3)Å, φ(2) = 23.08 (5)° and θ= 129.07 (4)° respectively. In most of the benzotheino ring systems the cyclohexyl ring adopts half-chair conformation (Akkurt *et al.*, 2008; Harrison *et al.*, 2006). The crystal structure is stabilized by two C—H···N intermolecular interactions. One of the C—H···N interaction links the molecules into head-head centrosymmetric dimers corresponding to graph set notation *R*^2^_2_(8) (Bernstein *et al.*, 1995) (Fig 2), while the other C—H···N interaction interaction generates chain of molecules in a zig-zag tape like pattern along c axis (Fig 2). Additionally, the supramolecular assembly is further stabilized by π–π stacking interaction between the pyrimidine and phenyl rings. The C2—C4 (-*x* - 1,1 - *y*,1 - *z*) disposed at a distance of 3.445 (4)Å.

### Experimental

A solution of 2-Amino-4,5,6,7-tetrahydro-benzo[*b*]thiophene-3-carbonitrile (1.78 g, 10 mmole) in triethylorthoformate (12 ml) was heated under reflux for 18 h; excess triethylorthoformate was removed under pressure. The residue was treated with petroleum ether. Solid that separated was filtered and recrystallized with petroleum ether to afford light brown crystals of *N*-(3-Cyano-4, 5, 6, 7-tetrahydro-benzo[*b*]thiophen-2-yl)-formimidic acid ethyl ester. 0.234 g, 1 mmole of this mixture and benzoic acid hydrazide (0.136 g, 1 mmole) was stirred at room temperature in toluene (5 ml) and then AcOH (0.06 g,1 mmole) was added and refluxed further till the completion of the reaction. The reaction mixture was then washed with water and dried over sodium sulfate. Toluene was removed under pressure to get analytically pure product. Yield 74%; mp; 196–198° C.

### Refinement

The H atoms were placed at calculated positions in the riding model approximation with aromatic C—H = 0.97 Å, heterocyclic C—H = 0.93 Å, and *U*_iso_(H) = 1.2*U*_eq_(N/C).

### Crystal data

**Table:**

C_17_H_14_N_4_S	*F*(000) = 640
*M**_r_* = 306.38	*D*_x_ = 1.443 Mg m^−^^3^
Monoclinic, *P*2_1_/*c*	Mo *K*α radiation, λ = 0.71073 Å
Hall symbol: -P 2ybc	Cell parameters from 3042 reflections
*a* = 8.6239 (16) Å	θ = 2.6–27.0°
*b* = 20.512 (4) Å	µ = 0.23 mm^−^^1^
*c* = 8.5952 (16) Å	*T* = 296 K
β = 111.975 (3)°	Block, white
*V* = 1410.0 (5) Å^3^	0.18 × 0.16 × 0.16 mm
*Z* = 4	

### Data collection

**Table:**

Bruker SMART APEX CCD detector diffractometer	3042 independent reflections
Radiation source: fine-focus sealed tube	2345 reflections with *I* > 2σ(*I*)
graphite	*R*_int_ = 0.057
ω scans	θ_max_ = 27.0°, θ_min_ = 2.6°
Absorption correction: multi-scan (*SADABS*; Bruker, 1998)	*h* = −10→11
*T*_min_ = 0.960, *T*_max_ = 0.964	*k* = −26→19
8272 measured reflections	*l* = −10→10

### Refinement

**Table:**

Refinement on *F*^2^	Primary atom site location: structure-invariant direct methods
Least-squares matrix: full	Secondary atom site location: difference Fourier map
*R*[*F*^2^ > 2σ(*F*^2^)] = 0.060	Hydrogen site location: inferred from neighbouring sites
*wR*(*F*^2^) = 0.202	H-atom parameters constrained
*S* = 1.25	*w* = 1/[σ^2^(*F*_o_^2^) + (0.1054*P*)^2^] where *P* = (*F*_o_^2^ + 2*F*_c_^2^)/3
3042 reflections	(Δ/σ)_max_ = 0.001
199 parameters	Δρ_max_ = 0.72 e Å^−^^3^
0 restraints	Δρ_min_ = −0.66 e Å^−^^3^

### Special details

**Table:**

Geometry. All s.u.'s (except the s.u. in the dihedral angle between two l.s. planes) are estimated using the full covariance matrix. The cell s.u.'s are taken into account individually in the estimation of s.u.'s in distances, angles and torsion angles; correlations between s.u.'s in cell parameters are only used when they are defined by crystal symmetry. An approximate (isotropic) treatment of cell s.u.'s is used for estimating s.u.'s involving l.s. planes.
Refinement. Refinement of *F*^2^ against ALL reflections. The weighted *R*-factor *wR* and goodness of fit *S* are based on *F*^2^, conventional *R*-factors *R* are based on *F*, with *F* set to zero for negative *F*^2^. The threshold expression of *F*^2^ > 2σ(*F*^2^) is used only for calculating *R*-factors(gt) *etc*. and is not relevant to the choice of reflections for refinement. *R*-factors based on *F*^2^ are statistically about twice as large as those based on *F*, and *R*- factors based on ALL data will be even larger.

### Fractional atomic coordinates and isotropic or equivalent isotropic displacement parameters (Å^2^)

**Table:**

	*x*	*y*	*z*	*U*_iso_*/*U*_eq_	
C1	−0.1145 (3)	0.57323 (12)	0.5033 (3)	0.0166 (6)	
C2	−0.2370 (3)	0.52330 (12)	0.4477 (3)	0.0160 (6)	
C3	−0.4107 (3)	0.44589 (13)	0.3912 (3)	0.0163 (6)	
C4	−0.5006 (3)	0.38839 (13)	0.4128 (3)	0.0177 (6)	
C5	−0.3157 (3)	0.56670 (13)	0.1641 (4)	0.0205 (6)	
H5	−0.3857	0.5635	0.0517	0.025*	
C6	−0.1062 (3)	0.61456 (13)	0.3786 (4)	0.0189 (6)	
C7	0.0023 (3)	0.58943 (13)	0.6675 (3)	0.0174 (6)	
C8	0.0944 (3)	0.64270 (13)	0.6629 (3)	0.0185 (6)	
C9	0.2214 (4)	0.67497 (14)	0.8139 (4)	0.0228 (6)	
H9A	0.2104	0.7220	0.8025	0.027*	
H9B	0.3332	0.6631	0.8226	0.027*	
C10	0.1936 (4)	0.65306 (15)	0.9725 (4)	0.0297 (7)	
H10A	0.2879	0.6667	1.0710	0.036*	
H10B	0.0940	0.6739	0.9760	0.036*	
C11	0.1742 (4)	0.57928 (15)	0.9757 (4)	0.0292 (7)	
H11A	0.1642	0.5667	1.0803	0.035*	
H11B	0.2735	0.5586	0.9710	0.035*	
C12	0.0201 (4)	0.55527 (13)	0.8282 (3)	0.0197 (6)	
H12A	0.0296	0.5087	0.8144	0.024*	
H12B	−0.0793	0.5629	0.8529	0.024*	
C13	−0.6266 (3)	0.35972 (13)	0.2766 (4)	0.0187 (6)	
H13	−0.6562	0.3776	0.1699	0.022*	
C14	−0.7074 (4)	0.30477 (14)	0.3003 (4)	0.0216 (6)	
H14	−0.7920	0.2862	0.2091	0.026*	
C15	−0.6648 (4)	0.27684 (14)	0.4573 (4)	0.0213 (6)	
H15	−0.7186	0.2393	0.4715	0.026*	
C16	−0.5407 (4)	0.30559 (15)	0.5931 (4)	0.0232 (7)	
H16	−0.5118	0.2875	0.6994	0.028*	
C17	−0.4598 (3)	0.36091 (13)	0.5716 (4)	0.0211 (6)	
H17	−0.3773	0.3800	0.6638	0.025*	
N1	−0.3331 (3)	0.52294 (11)	0.2782 (3)	0.0170 (5)	
N2	−0.4455 (3)	0.47305 (11)	0.2407 (3)	0.0190 (5)	
N3	−0.2852 (3)	0.47484 (10)	0.5218 (3)	0.0173 (5)	
N4	−0.2022 (3)	0.61279 (11)	0.2114 (3)	0.0202 (5)	
S1	0.04561 (9)	0.67369 (3)	0.46182 (9)	0.0205 (3)	

### Atomic displacement parameters (Å^2^)

**Table:**

	*U*^11^	*U*^22^	*U*^33^	*U*^12^	*U*^13^	*U*^23^
C1	0.0188 (14)	0.0123 (13)	0.0200 (14)	0.0057 (10)	0.0089 (11)	0.0046 (10)
C2	0.0157 (14)	0.0168 (13)	0.0145 (13)	0.0021 (10)	0.0044 (11)	−0.0027 (10)
C3	0.0148 (13)	0.0135 (13)	0.0187 (14)	0.0013 (10)	0.0039 (11)	−0.0001 (10)
C4	0.0193 (14)	0.0158 (13)	0.0182 (14)	0.0036 (11)	0.0072 (11)	0.0012 (11)
C5	0.0211 (15)	0.0189 (14)	0.0198 (15)	−0.0025 (11)	0.0057 (12)	0.0000 (11)
C6	0.0203 (15)	0.0161 (13)	0.0201 (15)	0.0041 (11)	0.0075 (12)	0.0011 (11)
C7	0.0173 (14)	0.0171 (14)	0.0169 (14)	0.0017 (11)	0.0055 (11)	−0.0026 (11)
C8	0.0173 (14)	0.0144 (13)	0.0216 (15)	0.0029 (11)	0.0048 (12)	−0.0019 (11)
C9	0.0213 (15)	0.0206 (15)	0.0240 (16)	−0.0038 (11)	0.0058 (12)	−0.0015 (11)
C10	0.0335 (18)	0.0271 (16)	0.0208 (16)	−0.0066 (14)	0.0015 (14)	−0.0032 (13)
C11	0.0303 (18)	0.0312 (17)	0.0210 (16)	−0.0031 (14)	0.0037 (14)	0.0018 (13)
C12	0.0223 (15)	0.0164 (14)	0.0181 (14)	−0.0019 (11)	0.0049 (12)	−0.0027 (11)
C13	0.0188 (15)	0.0186 (14)	0.0174 (14)	−0.0007 (11)	0.0054 (11)	−0.0009 (11)
C14	0.0193 (15)	0.0258 (15)	0.0204 (15)	−0.0018 (12)	0.0081 (12)	−0.0041 (12)
C15	0.0245 (15)	0.0165 (14)	0.0254 (16)	0.0010 (11)	0.0123 (13)	0.0011 (11)
C16	0.0215 (16)	0.0263 (16)	0.0201 (15)	−0.0030 (12)	0.0059 (12)	0.0042 (12)
C17	0.0174 (15)	0.0238 (15)	0.0194 (15)	−0.0007 (12)	0.0039 (12)	0.0028 (12)
N1	0.0164 (12)	0.0163 (11)	0.0172 (12)	−0.0049 (9)	0.0047 (10)	−0.0015 (9)
N2	0.0189 (12)	0.0180 (12)	0.0182 (12)	−0.0019 (9)	0.0048 (10)	−0.0020 (9)
N3	0.0186 (12)	0.0141 (11)	0.0192 (12)	0.0012 (9)	0.0071 (10)	0.0012 (9)
N4	0.0206 (13)	0.0204 (12)	0.0178 (12)	0.0017 (10)	0.0050 (10)	0.0032 (9)
S1	0.0209 (4)	0.0173 (4)	0.0224 (4)	−0.0018 (3)	0.0071 (3)	0.0017 (3)

### Geometric parameters (Å, °)

**Table:**

C1—C6	1.389 (4)	C9—H9A	0.9700
C1—C2	1.420 (4)	C9—H9B	0.9700
C1—C7	1.433 (4)	C10—C11	1.524 (4)
C2—N3	1.329 (3)	C10—H10A	0.9700
C2—N1	1.380 (3)	C10—H10B	0.9700
C3—N2	1.336 (4)	C11—C12	1.535 (4)
C3—N3	1.369 (3)	C11—H11A	0.9700
C3—C4	1.461 (4)	C11—H11B	0.9700
C4—C17	1.395 (4)	C12—H12A	0.9700
C4—C13	1.395 (4)	C12—H12B	0.9700
C5—N4	1.311 (4)	C13—C14	1.380 (4)
C5—N1	1.379 (3)	C13—H13	0.9300
C5—H5	0.9300	C14—C15	1.383 (4)
C6—N4	1.365 (4)	C14—H14	0.9300
C6—S1	1.731 (3)	C15—C16	1.386 (4)
C7—C8	1.360 (4)	C15—H15	0.9300
C7—C12	1.504 (4)	C16—C17	1.380 (4)
C8—C9	1.503 (4)	C16—H16	0.9300
C8—S1	1.739 (3)	C17—H17	0.9300
C9—C10	1.537 (4)	N1—N2	1.363 (3)
			
C6—C1—C2	115.3 (3)	H10A—C10—H10B	108.0
C6—C1—C7	113.5 (2)	C10—C11—C12	111.8 (3)
C2—C1—C7	131.2 (2)	C10—C11—H11A	109.3
N3—C2—N1	109.3 (2)	C12—C11—H11A	109.3
N3—C2—C1	134.9 (3)	C10—C11—H11B	109.3
N1—C2—C1	115.8 (2)	C12—C11—H11B	109.3
N2—C3—N3	115.8 (2)	H11A—C11—H11B	107.9
N2—C3—C4	121.5 (2)	C7—C12—C11	111.6 (2)
N3—C3—C4	122.7 (2)	C7—C12—H12A	109.3
C17—C4—C13	118.9 (2)	C11—C12—H12A	109.3
C17—C4—C3	119.9 (3)	C7—C12—H12B	109.3
C13—C4—C3	121.2 (3)	C11—C12—H12B	109.3
N4—C5—N1	121.0 (3)	H12A—C12—H12B	108.0
N4—C5—H5	119.5	C14—C13—C4	119.9 (3)
N1—C5—H5	119.5	C14—C13—H13	120.1
N4—C6—C1	127.6 (3)	C4—C13—H13	120.1
N4—C6—S1	121.4 (2)	C15—C14—C13	121.2 (3)
C1—C6—S1	111.0 (2)	C15—C14—H14	119.4
C8—C7—C1	111.1 (2)	C13—C14—H14	119.4
C8—C7—C12	122.8 (2)	C14—C15—C16	119.0 (3)
C1—C7—C12	126.1 (2)	C14—C15—H15	120.5
C7—C8—C9	125.0 (3)	C16—C15—H15	120.5
C7—C8—S1	113.3 (2)	C17—C16—C15	120.5 (3)
C9—C8—S1	121.7 (2)	C17—C16—H16	119.8
C8—C9—C10	109.4 (2)	C15—C16—H16	119.8
C8—C9—H9A	109.8	C16—C17—C4	120.5 (3)
C10—C9—H9A	109.8	C16—C17—H17	119.7
C8—C9—H9B	109.8	C4—C17—H17	119.7
C10—C9—H9B	109.8	N2—N1—C5	125.3 (2)
H9A—C9—H9B	108.2	N2—N1—C2	110.3 (2)
C11—C10—C9	111.2 (3)	C5—N1—C2	124.5 (2)
C11—C10—H10A	109.4	C3—N2—N1	101.6 (2)
C9—C10—H10A	109.4	C2—N3—C3	103.0 (2)
C11—C10—H10B	109.4	C5—N4—C6	115.7 (2)
C9—C10—H10B	109.4	C6—S1—C8	91.09 (13)
			
C6—C1—C2—N3	179.6 (3)	C3—C4—C13—C14	178.9 (2)
C7—C1—C2—N3	−1.3 (5)	C4—C13—C14—C15	−0.6 (4)
C6—C1—C2—N1	−0.9 (3)	C13—C14—C15—C16	1.2 (4)
C7—C1—C2—N1	178.1 (3)	C14—C15—C16—C17	−0.6 (4)
N2—C3—C4—C17	179.3 (2)	C15—C16—C17—C4	−0.5 (4)
N3—C3—C4—C17	0.2 (4)	C13—C4—C17—C16	1.1 (4)
N2—C3—C4—C13	−0.1 (4)	C3—C4—C17—C16	−178.3 (3)
N3—C3—C4—C13	−179.3 (2)	N4—C5—N1—N2	−179.1 (2)
C2—C1—C6—N4	0.6 (4)	N4—C5—N1—C2	0.3 (4)
C7—C1—C6—N4	−178.7 (3)	N3—C2—N1—N2	−0.4 (3)
C2—C1—C6—S1	179.33 (19)	C1—C2—N1—N2	−180.0 (2)
C7—C1—C6—S1	0.1 (3)	N3—C2—N1—C5	−179.9 (2)
C6—C1—C7—C8	0.5 (3)	C1—C2—N1—C5	0.5 (4)
C2—C1—C7—C8	−178.5 (3)	N3—C3—N2—N1	0.3 (3)
C6—C1—C7—C12	178.1 (2)	C4—C3—N2—N1	−179.0 (2)
C2—C1—C7—C12	−1.0 (5)	C5—N1—N2—C3	179.5 (2)
C1—C7—C8—C9	176.4 (2)	C2—N1—N2—C3	0.1 (3)
C12—C7—C8—C9	−1.2 (4)	N1—C2—N3—C3	0.5 (3)
C1—C7—C8—S1	−1.0 (3)	C1—C2—N3—C3	180.0 (3)
C12—C7—C8—S1	−178.6 (2)	N2—C3—N3—C2	−0.5 (3)
C7—C8—C9—C10	−17.3 (4)	C4—C3—N3—C2	178.7 (2)
S1—C8—C9—C10	159.9 (2)	N1—C5—N4—C6	−0.7 (4)
C8—C9—C10—C11	47.7 (3)	C1—C6—N4—C5	0.3 (4)
C9—C10—C11—C12	−62.6 (4)	S1—C6—N4—C5	−178.4 (2)
C8—C7—C12—C11	−11.0 (4)	N4—C6—S1—C8	178.3 (2)
C1—C7—C12—C11	171.7 (3)	C1—C6—S1—C8	−0.5 (2)
C10—C11—C12—C7	42.0 (3)	C7—C8—S1—C6	0.9 (2)
C17—C4—C13—C14	−0.6 (4)	C9—C8—S1—C6	−176.6 (2)

### Hydrogen-bond geometry (Å, °)

**Table:**

*D*—H···*A*	*D*—H	H···*A*	*D*···*A*	*D*—H···*A*
C5—H5···N2^i^	0.93	2.50	3.413 (3)	166
C11—H11B···N2^ii^	0.97	2.82	3.653 (5)	144

Symmetry codes: (i) −*x*−1, −*y*+1, −*z*; (ii) −*x*, −*y*+1, −*z*+1.
